# COVID-19: Post-recovery Manifestations

**DOI:** 10.7759/cureus.36886

**Published:** 2023-03-29

**Authors:** Safia Shaikh, Zunaira Siddiqi, Crystal Ukachukwu, Zainab Mehkari, Sadaf Khan, Ketan Pamurthy, Farhat Jahan, Amaiya Brown

**Affiliations:** 1 Clinical Research, Southwest Family Medicine Associates, Dallas, USA; 2 General Medicine, Dow University of Health Sciences, Civil Hospital Karachi, Karachi, PAK; 3 General Medicine, University College Hospital, Ibadan, NGA; 4 Internal Medicine, California Institute of Behavioral Neurosciences & Psychology, Fairfield, USA; 5 Research Approval, PPD Inc, Houston, USA; 6 Medical School, Brown University, Providence, USA; 7 Research, St. Michael Hospital Research Laboratory, Ontario, CAN; 8 Medical School, University of Arlington, Arlington, USA

**Keywords:** post-covid-19 recovery symptoms, post-covid-19 syndrome, covid-19, covid-19 manifestation, long covid

## Abstract

Background

Post-COVID-19 syndrome, also known as long COVID, is a disorder that has many characteristics, one of which is chronic fatigue following acute infection with the SARS-CoV-2 virus.

Methodology

We distributed a web-based survey among patients diagnosed with COVID-19 across the world and collected 190 responses regarding their demographics, histories, COVID-19 infection courses, and common symptoms.

Results

We found that about 85.3% of the patients experienced some form of symptom following recovery from the infection. Among the reported symptoms, 59% of patients experienced fatigue or lethargy, 48.9% reported decreased stamina, 32.6% reported shortness of breath, 16.8% had a persistent cough, and 23.7% experienced anxiety following recovery from COVID-19.

Conclusions

Reported symptoms closely resembled myalgic encephalomyelitis/chronic fatigue syndrome (ME/CFS); however, a deeper biochemical understanding of ME/CFS is required to confirm causation.

## Introduction

“Listen to your patient. The patient is telling you the diagnosis.” - Dr. Anthony Komaroff.

COVID-19 has become the greatest topic of research today due to the global havoc it has created and the constantly evolving modalities that it continues to present. Researchers are working hard to study the different effects that this positive-sense, single-stranded RNA virus (SARS-CoV-2) has on the health of the people it afflicts. As of September 26, 2021, according to data from Johns Hopkins University, there were 231,182,195 cases worldwide with 4,747,800 deaths [1]. Apart from the course that this virus takes during the acute infection phase, long-term clinical manifestations can leave patients debilitated and fatigued, with worsened quality of life. This chronic phase, termed long COVID, often presents with symptoms similar to myalgic encephalomyelitis/chronic fatigue syndrome (ME/CFS) [2].

The National Academies of Sciences, Engineering, and Medicine (NASEM) defines ME/CFS, also called systemic exertional intolerance disease (SEID), [3] as a persistent period of six months or longer characterized by fatigue, unrefreshing sleep, cognitive impairment, inability to perform pre-illness activities, and the worsening of symptoms following stress/exertion [4]. In 2015, it was estimated that between 836,000 and 2.5 million Americans suffered from ME/CFS, but recent studies are highlighting the variety in span and severity of the disorder’s manifestations, along with a possible link to COVID-19 [2]. One such study performed by Irish researchers followed 128 polymerase chain reaction (PCR)-diagnosed SARS-CoV-2 patients. Ten weeks after the initial symptoms, 52% reported persistent fatigue with no association linked to the severity of COVID-19, inflammatory markers, or proinflammatory molecules [2]. Another seven-month cohort study was conducted with 3,762 patients from 56 countries. After six months, the most commonly reported symptoms were fatigue (77.9%), post-exertional malaise (71.2%), and cognitive dysfunction (56.8%), with 67.5% requiring a reduced work schedule [2].

Our team of professionals conducted a web-based international survey focused on COVID-19 symptoms and post-COVID-19 manifestations, including fatigue, myalgia, brain fog, cognitive dysfunction, and persistent cough. This study highlights the importance of following patients entering the long COVID phase of symptoms to treat them appropriately and improve their quality of life.

Pathophysiology

CFS is a unique disorder in that it is often a multisystem disorder, causing direct damage to major organs such as the kidney, heart, and lungs [2]. However, there are several other postulations that have been made about the pathogenesis of post-COVID-19 ME/CFS. One such theory regards neuroinflammation. Inflammation triggers the release of inflammatory molecules, such as cytokines, prostaglandins, and complement factors, which can alter the activity of neurotransmitters when in the brain. This effect on neurochemistry can present as fatigue and decreased stamina in some individuals [5-7]. Another cause might be mitochondrial issues causing oxidative stress, an imbalance between body levels of reactive oxygen species (ROS) and antioxidants [8]. Yet another potential cause of ME/CFS is that COVID-19-induced damage to olfactory sensory neurons can cause a reduced outflow of cerebrospinal fluid (CSF) through the cribriform plate [9]. Nevertheless, there is still a large degree of uncertainty regarding the specifics of how the body is affected by ME/CFS from a biochemical standpoint. More studies need to be conducted to better understand the pathophysiology behind ME/CFS to address these lingering symptoms and provide a better quality of life to those suffering from them.

## Materials and methods

This study was conducted by a multidisciplinary team of professionals in response to a survey conducted over the last 18 months regarding the COVID-19 disease course and post-recovery manifestations. The survey was distributed in the United States, Pakistan, and Nigeria. The survey was randomly distributed through a Google Forms link among patients diagnosed with COVID-19, and the responses were recorded on a voluntary basis. The questionnaire was in the English language, and no ethnic, age, or gender restriction was applied. The survey contained 23 items divided into sections about COVID-19 symptoms, hospitalization, and post-acute COVID-19 recovery. The questions had multiple-choice items for patients to choose from. The following consent language was attached to the survey: “COVID-19 is raging through America. As scientists continue to understand the behavior of this virus new discoveries are still coming to light. Even though the mortality rate is not as severe as at the beginning of this pandemic this virus continues to pose challenges even after the recovery. This study is being conducted to understand the various post-recovery manifestation in COVID-19 patients. This questionnaire contains precise and relevant multiple-choice questions, which will take a few minutes to complete. Your participation in this research project will help us make sense of these manifestations and will take us one step further in our understanding of COVID-19 through the gathered data This survey is completely voluntary. The information you provide is anonymous and will remain confidential. The data will only be used in summary statistics for academic research purposes. There is no financial compensation for your participation in this study, nor are there any risks or discomforts of any kind. If you understand and agree please proceed.”

The questionnaire can be found at: https://docs.google.com/forms/d/e/1FAIpQLSecHsH4cAyyIFa8dgmCEcqCD5PD6qgvxiynsR4XDQ6Y3-seZg/viewform?usp=sf_link.

This cross-sectional, web-based study was submitted to the Sterling Institutional Review Board for review. It was approved as exempt (approval number: 8513-SShaikh). There are no conflicts of interest to disclose.

## Results

We collected data from an online survey of 190 patients globally (with a bias toward the United States, Pakistan, and Nigeria) who contracted COVID-19 and were 5-70 years old. Google for statistical analysis was considered while compiling the results for this study. Out of 190 patients, 68.9% were female, and 28.9% were male, with 2.1% preferring not to mention their gender identity.

Most patients were diagnosed with COVID-19 by positive nasal swab PCR test (75.5%), with the next largest group being diagnosed by positive rapid test (9%), followed by a doctor’s assessment based on acute symptoms, or antibody test (5.2% each).

**Figure 1:**
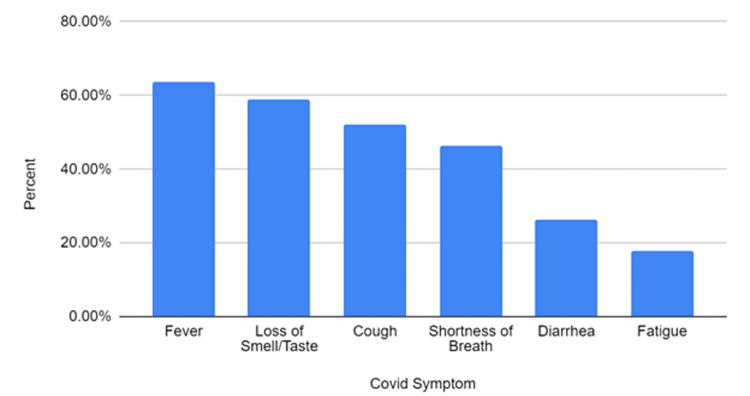
Acute-phase COVID-19 symptoms.

The most common symptoms were fever (63.7%), loss of smell/taste (58.9%), cough (52.1%), shortness of breath/difficulty breathing (46.3%), diarrhea (26.3%), and fatigue (17.9%). Other less common symptoms (occurring in 2% or less of the patients) were headache, brain fog, chills, lethargy, palpitations, weak memory, sore throat, dizziness, numbness, tingling, weakness, sinus/nasal congestion, body soreness, myalgia, rhinorrhea, muscle and joint pain, skin hypersensitivity, loss of appetite and laryngitis, as depicted in Figure 1.

**Figure 2:**
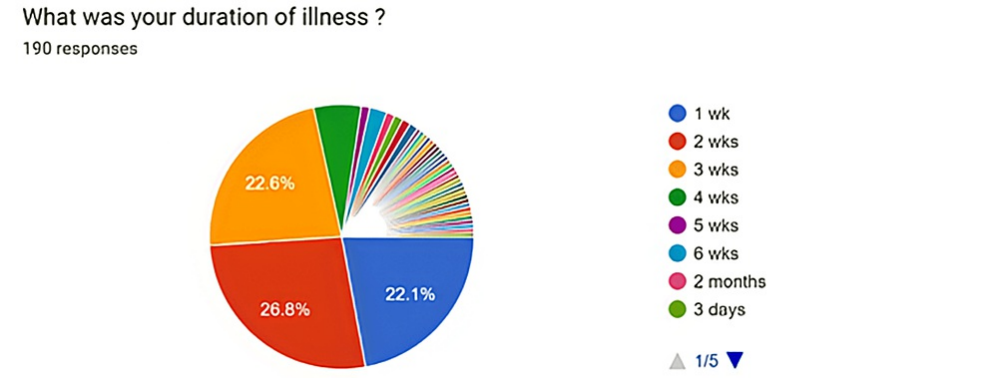
Duration of illness.

The majority of patients (78.9%) recovered from the acute phase within three weeks of disease onset. Among them, 22.1% recovered within one week, 26.8% within two weeks, and 22.6% within three weeks, as shown in Figure 2.

While 69.5% of the patients were healthy without any underlying comorbidity, the remaining 30.5% of patients had underlying conditions mainly diabetes mellitus (4.2%), asthma (7.9%), obesity (15.8%), and hypertension (8.9%). Over 96% of the patients were non-smokers. Overall, 75% of patients who reported no post-recovery symptoms also reported no comorbidities (Table 1).

**Table 1:** Comorbidities and tobacco exposure versus healthy subjects.

Comorbidities vs. healthy (Total population percentage)	
Healthy	69.5%
Diabetes mellitus	4.2%
Asthma	7.9%
Obesity	15.8%
Hypertension	8.9%
Smokers	<4%

Only 13.7% were hospitalized. Among those hospitalized, 46.2% required supplemental oxygen, 11.5% received plasma therapy, 23.1% were admitted to the intensive care unit, and only 3.8% required a ventilator.

Patients who did not require hospitalization either did not receive any treatment or received symptomatic treatment from their doctors (panadol, azithromycin, hydroxychloroquine, vitamin supplements, albuterol, etc.).

**Figure 3:**
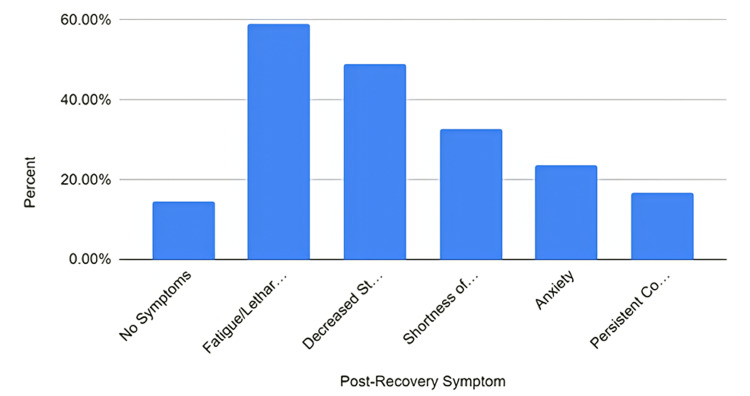
Post-recovery symptoms.

**Figure 4:**
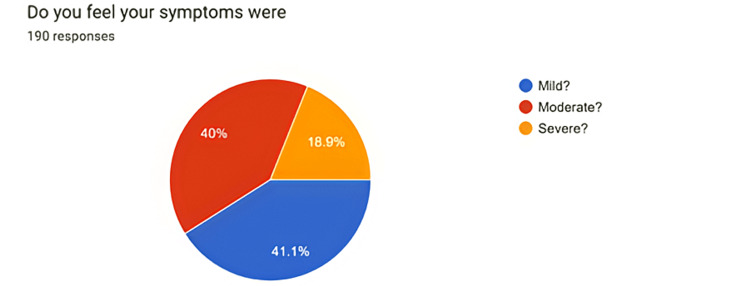
Severity of symptoms.

Patients had a wide range of symptoms reported post-recovery, with 14.7% reporting no symptoms, 26.4% reporting mild symptoms, 40% reporting moderate symptoms, and 18.9% reporting severe symptoms based on symptomology. Our study found that the most common post-recovery symptoms were fatigue/lethargy (58.9%), decreased stamina (48.9%), shortness of breath (32.6%), anxiety (23.7%), and persistent cough (16.8%) (Figure 3). Other reported symptoms included tachycardia, dizziness, and muscle weakness, especially with activity. All symptoms experienced were also categorized into mild (41.1%), moderate (40%), and severe (18.9%), as shown in Figure 4.

Doctors prescribed medication to resolve post-recovery symptoms for only 30.1% of patients who reported experiencing post-recovery symptoms. Overall, 21.4% of patients reported re-experiencing acute-phase COVID-19 symptoms days or weeks after recovery; however, only 7.8% were re-tested, and 30.8% of those patients tested positive.

## Discussion

Although several studies have explored the epidemiology, pathogenesis, and potential treatment of COVID-19, a lot is left to be said about the disease’s post-recovery manifestations and their treatments. This study investigated the course of the disease and various symptoms experienced by patients after recovery. In addition, to date, many studies have been conducted in individual countries and have a slight male predilection, but no major sex predilection [9-11]. On the other hand, this study cuts across several countries (primarily the United States, Pakistan, and the United Kingdom), with 67.4% of the surveyed participants identifying as women.

We identified fever, cough, shortness of breath, anosmia, dysgeusia, and diarrhea as the prevalent symptoms of COVID-19, which is consistent with previous studies [12]. While our study did include patients who reported comorbidities such as obesity, hypertension, asthma, and diabetes mellitus, the incidence was fairly low (30.5%), possibly due to the preponderance of young to middle-aged patients in this study.

Various diagnostic methods have been employed in the diagnosis of COVID-19. This study showed that a nasal swab PCR test was the modal diagnostic method (75.5%). This is not surprising as rapid diagnostic tests have played a crucial role in ensuring early diagnosis and prompt intervention (contact tracing, isolation, and supportive treatment) geared toward pandemic control. Other reported diagnostic methods included antibody assays, clinical diagnoses, and self-diagnosis.

Due to the rapid emergence of the pandemic at the time of questionnaire collection, treatment protocols were largely experimental, repurposing the already existing medications, such as hydroxychloroquine and remdesivir, for the treatment of COVID-19. Treatment measures varied widely among respondents in this study. Most respondents were managed on an outpatient basis, requiring neither oxygen therapy, convalescent plasma therapy, intensive care unit admission nor ventilatory support. Watchful waiting and supportive treatment (vitamin C, zinc, analgesics, hydration, bed rest, etc.) were the mainstays of treatment but some respondents received antibiotics and antimalarials (hydroxychloroquine), ivermectin, and/or remdesivir (6.8%).

Available evidence suggests that social habits such as tobacco smoking in patients with COVID-19 are associated with worse severity and poorer outcomes [13]. Almost all (>96%) of the respondents in our study did not report a history of smoking tobacco and, in keeping with the above, experienced mild-to-moderate disease courses and outcomes.

Despite the resolution of the disease, evidenced by negative COVID-19 tests, 21.4% of respondents experienced symptoms such as fatigue, lethargy, malaise, new-onset difficulty with breathing, anxiety, and joint aches from two weeks to as much as three months post-recovery. This rate of ME/CFS-like symptoms post-COVID-19 recovery agrees with similar studies that estimated that post-COVID-19 syndrome occurs in 10-35% of patients [14].

This phenomenon has been nicknamed long haul or long COVID and is defined as “signs and symptoms developed during or following a disease consistent with COVID-19 and which continue for more than four weeks but are not explained by alternative diagnoses.” However, the National Institute for Health and Care Excellence (NICE) recommends reservation of the term “post-covid syndrome” for symptoms occurring 12 weeks after infection [15].

Interestingly, this chronic post-viral syndrome has been previously reported following an outbreak of SARS coronavirus in Southeast Asia in 2003. That outbreak’s post-acute manifestations were characterized by fatigue, non-specific myalgia, depression, and other symptoms such as those experienced by patients with ME/CFS [16]. This is also not specific to the coronaviruses, as post-infectious fatigue syndromes have been observed in association with the Ebola virus, dengue virus, enteroviruses, bacteria such as *Borrelia burgdorferi* and *Mycoplasma pneumoniae*, and even parasites such as *Giardia lamblia* [2].

ME/CFS caused by long COVID is a somewhat difficult disorder to diagnose because there is currently no biomarker test or precise diagnostic that can be used to identify ME/CFS in patients. Objective investigations such as pulmonary function tests, performed on patients with post-COVID-19 fatigue and dyspnea revealed reduced forced vital capacity, lower diffusing capacity of the lungs for carbon monoxide, and reduced six-minute walk test [17-19]. However, no additional testing or treatment measures were offered to affected respondents in this study.

Due to the lack of medical understanding, conclusive biochemical diagnostics, or treatments for ME/CFS, many patients remain undiagnosed for extended periods and suffer greatly as a result. Some patients even report hostility from physicians or treatments that exacerbate their symptoms [19]. Among patients in our study who reported experiencing post-COVID-19 symptoms, 65.9% were not prescribed medication by their doctors. With a greater understanding of the pathophysiology of the disease, a more legitimate diagnostic test might increase the accuracy of diagnoses and prevent significantly worsened quality of life in these patients.

## Conclusions

The effects of long COVID are far-reaching and debilitating and require an extended period of rehabilitation. While the duration of intensive care and use of mechanical ventilation during the acute phase of COVID-19 were identified as risk factors for ME/CFS, other risk factors are largely unknown. There does, however, appear to be a strong correlation between having no comorbidities and experiencing no post-COVID-19 symptoms. In addition, this study demonstrated a link between COVID-19 infection and fatigue, shortness of breath, and other chronic symptoms at a rate consistent with previous studies. While there seems to be a clear association between COVID-19 infection and symptoms characteristic of ME/CFS, more studies need to be performed on the biochemistry of ME/CFS to establish more testable criteria and allow for more accurate studies of this association.
